# Crises and complexity: how can we make health interventions succeed?

**DOI:** 10.1093/heapol/czaf032

**Published:** 2025-06-04

**Authors:** Zaruhi Mkrtchyan, Yolanda Moyo, Fiammetta Bozzani, Heather Ingold, Graham Medley, Sergio Torres-Rueda, Fern Terris-Prestholt, Lorna Guinness, Evaluation of Social and Population Health Interventions (ESPHI) Group, Alaa Aziz, Andres Madriz Montero, Anna M Foss, Anna Vassall, Cassandra Nemzoff, Catherine Pitt, David Bath, Fern Terris-Prestholt, Fiammetta Bozzani, Francis Ruiz, Graham Medley, Heather Ingold, Ian Yoon, Iris Mosweu, Jahanzaib Sohail, Joseph Kazibwe, Joseph Tankwa, Kathleen McGee, Katherine Snyman, Kenneth Katumba, Laura Lunani, Lebohang Letsela, Linda Sande, Lorna Benton, Lorna Guinness, Mamtuti Panneh, Maninder Pal Singh, Miriam Abdulla, Mitzy Gafos, Mohammad Almari, Nikita Arora, Olimpia Lamberti, Paoli Behanzin, Peach Indravudh, Pooja Shah, Sedona Sweeney, Sergio Torres-Rueda, Silke Fernandes, Takuya Yamanaka, Tara Beattie, Yolando Moyo, Zaruhi Mkrtchyan, Anna M Foss, Francis Ruiz

**Affiliations:** Department of Global Health and Development, London School of Hygiene and Tropical Medicine, Keppel Street, London WC1E 7TH, United Kingdom; Department of Global Health and Development, London School of Hygiene and Tropical Medicine, Keppel Street, London WC1E 7TH, United Kingdom; Department of Global Health and Development, London School of Hygiene and Tropical Medicine, Keppel Street, London WC1E 7TH, United Kingdom; Department of Global Health and Development, London School of Hygiene and Tropical Medicine, Keppel Street, London WC1E 7TH, United Kingdom; Department of Global Health and Development, London School of Hygiene and Tropical Medicine, Keppel Street, London WC1E 7TH, United Kingdom; Department of Global Health and Development, London School of Hygiene and Tropical Medicine, Keppel Street, London WC1E 7TH, United Kingdom; Genesis Analytics, Health Practice, 50 6th Road, Hyde Park, Johannesburg 2196, South Africa; Department of Global Health and Development, London School of Hygiene and Tropical Medicine, Keppel Street, London WC1E 7TH, United Kingdom; Department of Global Health and Development, London School of Hygiene and Tropical Medicine, Keppel Street, London WC1E 7TH, United Kingdom; Department of Global Health and Development, London School of Hygiene and Tropical Medicine, Keppel Street, London WC1E 7TH, United Kingdom; Department of Global Health and Development, London School of Hygiene and Tropical Medicine, Keppel Street, London WC1E 7TH, United Kingdom

**Keywords:** complex emergencies, decision-making, health policy, impact, interdisciplinary, international health policy, policy evaluation, policy implementation, policy research, research policy, cost-effectiveness

## Abstract

The end of the COVID-19 global health emergency presents an opportunity to reflect on actions needed to enhance the effectiveness of responses to any future shocks. We highlight critical areas requiring attention from researchers and research commissioners to enhance the identification and adoption of ‘good value’ interventions, and we discuss the complexities of evidence-informed decision-making across multiple sectors, the evolving role of modeling, and the need for improved stakeholder engagement and institutional coordination to effectively address interconnected health and policy challenges. We conclude the commentary by making a set of related recommendations to support intervention identification and implementation. Researchers, policymakers, and other key stakeholders should: renew efforts to step out of silos and to develop methods and frameworks that link and synthesize evidence from multiple sources and perspectives, to support planetary health goals and the ‘One Health’ concept; support more research into understanding the constraints to adopting interventions regarded as good value for money, to enhance evaluation methods ex ante and to better inform systems and stakeholders of the implementation requirements; and maintain an ongoing commitment to equitable research partnerships to ensure that evidence use is relevant for the target settings.

Key messages

- Recent global public health threats and crises have emphasized long-recognized perspectives on the interconnectedness between human, animal, and environmental health, wealth, and well-being, and on the structural factors that impact on progress in all these areas. Addressing challenges in evidence-informed policymaking impacting across multiple sectors is essential for preventing disease, reducing health inequalities, and ensuring global health security.
- Making decisions on appropriate interventions in this context is necessarily difficult and complex and involves a number of considerations, including the availability of evidence, guidance, the content and context of policies, opportunities for cross-sector collaboration, modeling, institutionalization of effective priority setting frameworks, and the need for equitable partnerships.
- Going forward, to support the effective identification and adoption of good value interventions, we recommend that researchers and research commissioners should:
  - move beyond narrow approaches to inquiry and instead apply multidisciplinary methods and frameworks as part of evidence generation for policy decisions;
  - gain better insight on the challenges affecting the adoption and implementation of cost-effective policies to enhance their uptake and impact; and
  - maintain a strong focus on equitable research partnerships to ensure that evidence is both locally owned and useful for the intended settings.

The end of COVID-19 as a global health emergency has led to much discussion on the implications for future pandemic preparedness and response, but it also offers an opportunity to reflect on resilience more broadly, and the actions needed to enhance the effectiveness of responses to any future shocks. This commentary aims to summarize areas that demand attention by researchers, policymakers, and research commissioners on the impact that increasing complexity and the growing risk of further public health emergencies will have on the identification and adoption of ‘good value’ interventions. Whilst recognizing the ultimately political nature of decision-making and priority setting (Goddard *et al.* 2006), we provide a number of recommendations that should inform an approach which from the outset recognizes the need for comprehensiveness and the breaking down of scientific silos in the pursuit of public health improvements.

Evidence creation is undoubtedly an important foundation in the development of effective policies. However, its mere existence does not in itself guarantee successful policy implementation. This is especially the case if the evidence base used lacks a multidisciplinary perspective and fails to account for factors influencing the capacity to benefit. For example, modeling and observational studies of COVID-19 predicted the effectiveness of testing or vaccination in controlling the spread of the virus and preventing hospitalization and mortality (Jahn *et al.* 2021, Shimizu *et al.* 2021, Andrews *et al.* 2022, Pageaud *et al.* 2022, Mallah *et al.* 2023, Smoll *et al.* 2023). In addition, WHO advice for policymakers and technical guidelines were also available (WHO 2024). Despite this, real-world implementation of these measures faced significant hurdles in terms of timeliness, resources, public compliance, and logistics, resulting in a level of achievement that varied substantially among countries, independent of their income levels (Zheng *et al.* 2021, Andrews *et al.* 2022, Homan *et al.* 2022, Mallah *et al.* 2023, Smoll *et al.* 2023).

Decision-makers need to manage multiple perspectives when considering evidence to inform policy. Factors such as evidence type, who produced it, where and when it was collected, matter. Evidence synthesis approaches for guideline development, for example, have been pragmatically adjusted, and not always consistently, to accommodate a range of evidence types addressing multiple aspects of importance (Ingold *et al.* 2024). Moreover, health systems themselves are complex adaptive systems characterized by high levels of interaction between their elements (Paina and Peters 2012). Within them, many actors play a role in influencing governments to adopt policy solutions, and their interaction in the policy space ultimately determines what is prioritized.

It has long been recognized that health is the result of a complex interplay between activities and outcomes within and outside of the health sector. The interface of climate change and health, and the fight against antimicrobial resistance, for example, bring further challenges, including the need for potentially more complex scientific methods for generating and synthesizing multisectoral evidence. In such instances, there is a need to expand stakeholder involvement in decision-making, and often as part of the research itself, to include *trans*-sectoral actors to address interconnected goals (Ramponi *et al.* 2024). Cross-cutting system strengthening may be needed to improve the impact of multiple interventions across human, animal, and environmental health (Ferraz and Barreira 2024).

Modeling has become an established technique to support decision-making, although as the COVID-19 pandemic has shown, policymaker and user engagement in the design and use of models has been patchy (Hadley *et al.* 2021). More efforts are needed to ensure that meaningful stakeholder engagement is embedded throughout modeling projects from inception to completion which will support their credibility and improve user uptake. In addition, models that aim to integrate information across disciplines (e.g. combined micro-/macroeconomic models and infectious disease transmission models; ‘model chains,’ such as those linking climate change to extreme weather patterns, and to health outcomes and costs) are likely to become more commonplace. This could also involve potential extensions to ‘whole health system’ models (Hallett *et al.* 2025) so that they include other sectors. In this context, understanding what best practice entails—both technically and in terms of supporting policy—will be critical. This highlights an enormous challenge around capacity building needs for more complex approaches, including appropriate model literacy, especially in resource constrained settings. Research should therefore also identify scenarios and opportunities to minimize the capacity requirements for analysis and interpretation (Nemzoff *et al.* 2023, Kuehn *et al.* 2024).

The need for exploring coordinated policy actions both within and outside the health sector entails further research on what is required for successful evidence-informed decision-making processes and institutional architectures. Typically implemented priority setting processes, such as Health Technology Assessment, will undoubtedly need further enhancement going forward, to consider environmental impacts (Hensher 2024), the ‘One Health’ approach, and the growing urgency for cross-government collaboration and coordination (Ramponi *et al.* 2024).

Decolonization and the goal of power decentralization with respect to research conduct and impact represent additional factors needing strengthening in any evidence-to-policy support effort. In this context, we define decolonization as the need to shift decision-making power and research priorities to local stakeholders. The legitimate locus of decision-making is local and should underpin the clear articulation of whose priorities are considered, in the support of evidence uptake. Meaningful collaboration between Global North and Global South researchers across various disciplines is more than ever a crucial requirement (Ryan *et al.* 2023, Khan *et al.* 2024), as demonstrated by the ALERRT epidemic research network (ALERRT 2016). However, we recognize that this may be even more challenging to achieve going forward given declining donor flows and an emerging antiscience sentiment that introduces controversy in areas particularly demanding of cross-sectoral action such as climate change (Pagel *et al.* 2024).

Based on the above, we recommend actions across three broad areas:

- ***Transcending individual disciplines*** There needs to be a renewed effort to step out of silos and to develop methods and frameworks linking evidence chains to support planetary health goals and the ‘One Health’ concept to confront potential future shocks. For example, London School of Hygiene and Tropical Medicine (LSHTM) has recently joined a newly established consortium involving climate modelers, epidemiologists, economists, and anthropologists working to improve policies and interventions to tackle the effects of climate change on diarrheal diseases (LSHTM 2024).
- ***Overcoming adoption hurdles*** Further research is necessary to better understand the constraints to adopting interventions that would otherwise be regarded as good value for money, both to improve the methods of evaluating such interventions ex ante and to better inform systems and stakeholders of the implementation requirements. Cross-sectoral economic evaluation approaches that respect opportunity costs of actual expenditures will be an important component of such research. In addition, the pattern of constraints in low-to-middle-income country contexts may be different or more severe compared to higher-income settings and therefore needs careful analysis when evaluating interventions.
- ***Equitable partnerships*** An ongoing commitment is maintained to equitable research partnerships to ensure that evidence use is relevant for the target settings, enhances local ownership, and builds on local capacity. This should leverage key recent developments to strengthen localized decision-making in the Global South such as the creation of the Africa Centres for Disease Control, which has a key role in supporting member states of the African Union with evidence-informed guidance. In addition, the Aurum Institute is another example of a Global South-led organization working to strengthen equitable partnerships, build capacity, and support the development of locally relevant evidence (AURUM 2025).

The multiple threats and crises currently being faced by public health systems and societies more generally have highlighted the long-recognized perspectives on the interconnectedness between human, animal, and environmental health, wealth, and well-being and on the structural factors that impact on progress in all these areas. We are at a pivotal moment to support innovation in research and policy that transcends usual boundaries to address challenges that have always been complex, and are only likely to get more so going forward.

## Members of ESPHI

Alaa Aziz, Andres Madriz Montero, Anna M. Foss, Anna Vassall, Cassandra Nemzoff, Catherine Pitt, David Bath, Fern Terris-Prestholt, Fiammetta Bozzani, Francis Ruiz, Graham Medley, Heather Ingold, Ian Yoon, Iris Mosweu, Jahanzaib Sohail, Joseph Kazibwe, Joseph Tankwa, Kathleen McGee, Katherine Snyman, Kenneth Katumba, Laura Lunani, Lebohang Letsela, Linda Sande, Lorna Benton, Lorna Guinness, Mamtuti Panneh, Maninder Pal Singh, Miriam Abdulla, Mitzy Gafos, Mohammad Almari, Nikita Arora, Olimpia Lamberti, Paoli Behanzin, Peach Indravudh, Pooja Shah, Sedona Sweeney, Sergio Torres-Rueda, Silke Fernandes, Takuya Yamanaka, Tara Beattie, Yolando Moyo, Zaruhi Mkrtchyan.

## Data Availability

No new data were generated or analysed in support of this research.

## Reflexivity statement

In writing this commentary, we aimed to ensure that the authors’ group was diverse and inclusive, comprising doctoral students and researchers at different levels of seniority. The team brings extensive experience in priority setting, modeling and analysis in both high-income and low- and middle-income settings. With expertise in various methods, the authors offer valuable insights from a variety of geographical contexts, ensuring that the issues discussed in this commentary are relevant across different settings.
